# Ciprofloxacin-Resistant *Salmonella enterica* Serotype Typhi, United States, 1999–2008

**DOI:** 10.3201/eid1706.100594

**Published:** 2011-06

**Authors:** Felicita Medalla, Maria Sjölund-Karlsson, Sanghyuk Shin, Emily Harvey, Kevin Joyce, Lisa Theobald, Benjamin L. Nygren, Gary Pecic, Kathryn Gay, Jana Austin, Andrew Stuart, Elizabeth Blanton, Eric D. Mintz, Jean M. Whichard, Ezra J. Barzilay

**Affiliations:** Author affiliations: Centers for Disease Control and Prevention, Atlanta, Georgia, USA (F. Medalla, M. Sjölund-Karlsson, K. Joyce, L. Theobald, B.L. Nygren, G. Pecic, K. Gay, J. Austin, A. Stuart, E. Blanton, E.D. Mintz, J.M. Whichard, E.J. Barzilay);; California Emerging Infections Program, Oakland, California, USA (S. Shin);; Massachusetts Department of Public Health, Jamaica Plain, Massachusetts, USA (E. Harvey)

**Keywords:** Salmonella Typhi, ciprofloxacin, antimicrobial drug resistance, fluoroquinolones, typhoid fever, bacteria, dispatch

## Abstract

We report 9 ciprofloxacin-resistant *Salmonella enterica* serotype Typhi isolates submitted to the US National Antimicrobial Resistance Monitoring System during 1999–2008. The first 2 had indistinguishable pulsed-field gel electrophoresis patterns and identical *gyrA* and *parC* mutations. Eight of the 9 patients had traveled to India within 30 days before illness onset.

Typhoid fever, caused by *Salmonella enterica* serotype Typhi, is a systemic bacterial illness that has been rare in the United States in the era of modern sanitation (*1**,**2*). However, typhoid fever remains common in many developing countries. In the United States, 72%–81% of patients with typhoid fever report international travel in the month before illness onset (*1**,**3**–**5*). Highest risk has been associated with travel to southern Asia (*1**–**5*).

Fluoroquinolones (e.g., ciprofloxacin) are frequently used to treat typhoid fever in adults (*4**,**6*). Ciprofloxacin resistance is rare; however, resistance to the quinolone nalidixic acid in the US National Antimicrobial Resistance Monitoring System (NARMS) increased from 19% of isolates tested in 1999 to 59% in 2008 (*7*). Nalidixic acid resistance in *S. enterica* serotype Typhi, which has been associated with overseas travel, particularly to southern Asia, correlates with decreased susceptibility to ciprofloxacin (MIC >0.12 µg/mL) (*4**–**6**,**8*). Increased risk for fluoroquinolone treatment failure has been demonstrated in *Salmonella* infections from strains with decreased susceptibility to ciprofloxacin (*6**,**8**,**9*). Chromosomal point mutations in the *gyrA* and *parC* topoisomerase genes are mechanisms of quinolone resistance in *Salmonella* spp. Other resistance mechanisms include efflux pumps, reduced outer membrane permeability, and plasmid-borne genes (e.g., *qnr*, *aac-6′-Ib-cr* genes) (*6**,**8**,**10**–**12*). We report 9 ciprofloxacin-resistant (MIC >4 µg/mL) *S. enterica* serotype Typhi isolates detected in the United States during 1999–2008.

## The Cases

State public health laboratories receive *Salmonella* isolates from clinical diagnostic laboratories as part of routine surveillance. State and local health department officials report demographic, clinical, and travel information about laboratory-confirmed typhoid fever on a standard form to the Centers for Disease Control and Prevention (CDC, Atlanta, GA, USA). Participating states began submitting all *S. enterica* serotype Typhi isolates to NARMS in 1999; since 2003, all state public health laboratories have participated. Isolates were tested for susceptibility by using broth microdilution (Sensititre; Trek Diagnostics, Westlake, OH, USA). MICs were determined for 15 antimicrobial agents and interpreted by using Clinical and Laboratory Standards Institute (CLSI) criteria when available (Table 1) (*7**,**13*). For ciprofloxacin-resistant isolates, subtyping by pulsed-field gel electrophoresis (PFGE) was performed by using the protocol established by the National Molecular Subtyping Network for Foodborne Disease Surveillance (PulseNet) (*14*). PFGE pattern similarity was assessed by cluster analysis (Dice, UPGMA [unweighted pair group method using arithmetic averages]) and band-matching applications of BioNumerics software (Applied Maths, Sint-Martens-Latem, Belgium) and confirmed by visual comparison (Figure). For ciprofloxacin-resistant isolates detected for 1999–2005, sequencing of the quinolone resistance–determining region (QRDR; defined as amino acids 67–106 for *gyrA*) was performed according to the methods described by Crump et al. (*6*), and additional patient information (e.g., antimicrobial drug treatment) was requested by using a questionnaire with institutional review board approval.

**Table 1:** MICs of antimicrobial agents tested for 9 ciprofloxacin-resistant *Salmonella enterica* serotype Typhi isolates detected in the National Antimicrobial Resistance Monitoring System, United States, 1999–2008

Antimicrobial class and agent*	MIC, µg/mL,* by patient no. (isolate)								
	Patient 1 (MA-03)	Patient 2† (CA-05)	Patient 3 (CA-06)	Patient 4 (TX-06)	Patient 5 (AZ-06)	Patient 6 (NY-07)	Patient 7 (CA-07)	Patient 8 (NJ-07)	Patient 9 (LAC-07)
Quinolones									
Ciprofloxacin	>4	>4	>4	>4	>4	>4	>4	>4	>4
Nalidixic acid	>32	>32	>32	>32	>32	>32	>32	>32	>32
Aminoglycosides									
Amikacin	<0.5	1	1	1	1	1	1	1	1
Gentamicin	<0.25	<0.25	<0.25	<0.25	<0.25	<0.25	<0.25	<0.25	<0.25
Kanamycin	<8	<8	<8	<8	<8	<8	<8	<8	<8
Streptomycin	<32	<32	<32	>64	<32	<32	<32	<32	<32
β-lactam–β-lactamase inhibitor									
Amoxicillin-clavulanic acid	<1/0.5	<1/0.5	<1/0.5	8/4	<1/0.5	<1/0.5	<1/0.5	<1/0.5	<1/0.5
Cephems									
Cefoxitin	4	4	4	4	2	4	4	4	4
Ceftiofur	0.5	0.5	0.5	0.5	0.25	0.5	0.5	0.5	0.5
Ceftriaxone	<0.25	<0.25	<0.25	<0.25	<0.25	<0.25	<0.25	<0.25	<0.25
Folate pathway inhibitors									
Sulfonamide‡	>512	>256	<16	>256	>256	<16	>256	<16	<16
Trimethoprim- sulfamethoxazole	>4/76	>4/76	<0.12/ 2.38	>4/76	>4/76	<0.12/ 2.38	>4/76	<0.12/ 2.38	<0.12/ 2.38
Penicillins									
Ampicillin	2	<1	<1	>32	<1	<1	<1	<1	<1
Phenicols									
Chloramphenicol	4	4	4	>32	4	4	4	4	4
Tetracyclines									
Tetracycline	>32	>32	<4	<4	>32	<4	>32	<4	<4

*Classes of antimicrobial agents defined by the Clinical and Laboratory Standards Institute (CLSI) were used to categorize agents (*7*,*13*). MICs were interpreted by using CLSI criteria when available (*7*,*13*): ciprofloxacin (resistance breakpoint, >4 µg/mL); nalidixic acid (>32); amikacin (>64); gentamicin (>16); kanamycin (>64); amoxicillin-clavulanic acid (>32/16); cefoxitin (>32); ceftiofur (>8); ceftriaxone (>4); sulfamethoxazole/sulfisoxazole (>512); trimethoprim-sulfamethoxazole (>4/76); ampicillin (>32); chloramphenicol (>32); and tetracycline (>16). For streptomycin, resistance was defined as MIC >64 µg/mL (*7*). If growth was not inhibited by the highest concentration of the agent in the panel, the MIC was reported as above the highest concentration. †Isolate was cultured from a blood specimen. Another isolate was cultured from fecal samples, which had MIC <0.5 µg/mL for amikacin and same MICs for other agents tested. ‡Sulfamethoxazole was used during 1999–2003 and sulfisoxazole since 2004 to represent sulfonamides.

**Figure:**
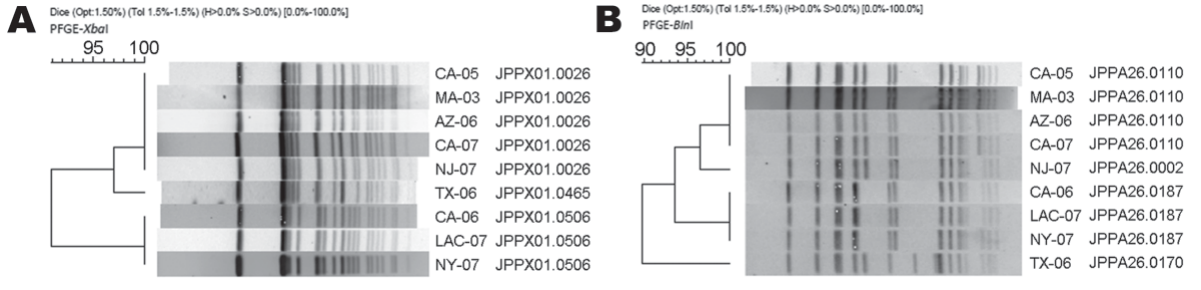
Pulsed-field gel electrophoresis (PFGE) *Xba*I (A) and *Bln*I (B) patterns of 9 ciprofloxacin-resistant *Salmonella enterica* serotype Typhi isolates detected in the National Antimicrobial Resistance Monitoring System, 1999–2008. PFGE pattern similarity was assessed by cluster analysis (Dice, UPGMA [unweighted pair group method using arithmetic average]) and band-matching applications of BioNumerics software (Applied Maths, Sint-Martens-Latem, Belgium) and confirmed by visual comparison. PulseNet only considers band markings found within the scale of the global standard, which are all bands between 20.5 kb and 1,135 kb. The cluster parameters are Dice coefficient and UPGMA with the tolerance of band position of 1.5% and optimization of 1.5%.

During 1999–2005, we detected 2 (0.1%) cases of ciprofloxacin resistance among 1,690 *S. enterica* serotype Typhi isolates. Case reports follow.

In 2003, a 1-year-old girl had onset of fever 1 day before arriving in the United States from India. A blood specimen collected 3 days after fever onset yielded *S. enterica* serotype Typhi. Diarrhea or vomiting at time of specimen collection was not reported. Information about antimicrobial drug treatment was not available. The child was hospitalized for 14 days.

In 2005, a 2-year-old girl had onset of diarrhea, which was treated with ofloxacin, 2 days before she arrived in the United States from India. Seven days later, she continued to have diarrhea, and fever, vomiting, and abdominal cramps developed. She was hospitalized and treated with antimicrobial agents, including ciprofloxacin. Blood and fecal specimens collected 3 weeks after illness onset yielded *S. enterica* serotype Typhi. The patient was discharged after 14 days of hospitalization. She had lived in India for 6 months before traveling to the United States.

The *S. enterica* serotype Typhi isolates were resistant to ciprofloxacin (Tables 1, 2) and had indistinguishable PFGE patterns when restriction enzymes *Xba*I and *Bln*I were used: PulseNet-designated *Xba*I pattern JPPX01.0026 and *Bln*I pattern JPPA26.0110 (Table 2; Figure). QRDR sequencing showed *gyrA* mutations resulting in a serine to tyrosine substitution at codon 83 and an aspartic acid to asparagine substitution at codon 87, and a *parC* mutation conferring a serine to isoleucine substitution at codon 80.

**Table 2:** Patient and isolate description, resistance to other antimicrobial agents, PFGE pattern, and travel reported for 9 ciprofloxacin-resistant *Salmonella enterica* serotype Typhi infections detected in the National Antimicrobial Resistance Monitoring System, United States, 1999–2008*

Patient no. (isolate)	Age, y/ sex	Site	Specimen collection year	Specimen source	Resistance to other agents	PFGE *Xba*I pattern†	PFGE *Bln*I pattern‡	Travel§
1 (MA-03)	1/F	MA	2003	Blood	Cot, Fis, Nal, Tet	JPPX01.0026	JPPA26.0110	India
2 (CA-05)	2/F	CA	2005	Blood	Cot, Fis, Nal, Tet	JPPX01.0026	JPPA26.0110	India
3 (CA-06)	26/F	CA	2006	Blood	Nal	JPPX01.0506	JPPA26.0187	India
4 (TX-06)	8/M	TX	2006	Blood	Amp, Chl, Cot, Fis, Nal, Str	JPPX01.0465	JPPA26.0170	India, other
5 (AZ-06)	5/M	AZ	2006	Stool	Cot, Fis, Nal, Tet	JPPX01.0026	JPPA26.0110	India
6 (NY-07)	6/M	NYC	2007	Stool	Nal	JPPX01.0506	JPPA26.0187	India
7 (CA-07)	22/M	CA	2007	Stool	Cot, Fis, Nal, Tet	JPPX01.0026	JPPA26.0110	India
8 (NJ-07)	28/M	NJ	2007	Blood	Nal	JPPX01.0026	JPPA26.0002	India
9 (LAC-07)	48/F	LAC	2007	Blood	Nal	JPPX01.0506	JPPA26.0187	Unknown

*PFGE, pulsed-field gel electrophoresis. State/local public health laboratories that submitted isolates: MA, Massachusetts; CA, California; TX, Texas; AZ, Arizona; NYC, New York City; NJ, New Jersey; LAC, Los Angeles County, California. Resistance to antimicrobial agents other than ciprofloxacin: Cot, trimethoprim–sulfamethoxazole; Fis, sulfamethoxazole or sulfisoxazole; Nal, nalidixic acid; Tet, tetracycline; Amp, ampicillin; Chl, chloramphenicol; Str, streptomycin. †National Molecular Subtyping Network for Foodborne Disease Surveillance (PulseNet)–designated PFGE patterns using restriction enzyme *Xba*I (data as of 2009 Oct 21): JPPX01.0026, the most common *Xba*I pattern among 3,233 isolates with reported *Xba*I pattern in PulseNet, was detected in 486 (15.0%); JPPX01.0465 and JPPX01.0506 each were detected in 12 (0.4%) isolates. ‡PulseNet-designated PFGE patterns using restriction enzyme *Bln*I (data as of 2009 Oct 21): JPPA26.0002, the most common *Bln*I pattern among 409 isolates with reported *Bln*I pattern in PulseNet, was detected in 61 (14.9%) isolates; JPPA26.0110 was detected in 5 (1.2%), JPPA26.0187 was detected in 3 (0.7%), and JPPA26.0170 was detected in 1 (0.2%). §Travel outside the United States reported in the 30 d before illness onset; patient 4 also traveled to Bangladesh and the United Arab Emirates.

Seven (0.6%) ciprofloxacin-resistant infections were detected among patients from whom 1,131 *S. enterica* serotype Typhi isolates were submitted during 2006–2008 (Table 2). The 7 cases occurred in 2006 and 2007. Patients were a median of 22 years of age (range 5–48 years); 5 (71%) were male. All 6 patients with known travel histories reported travel to India in the 30 days before illness onset. In addition to *Xba*I JPPX01.0026 and *Bln*I JPPA26.0110, 3 different *Xba*I and *Bln*I pattern combinations were detected in the 7 isolates (Table 2; Figure).

## Conclusions

We describe ciprofloxacin-resistant *S. enterica* serotype Typhi isolates from 9 patients in the United States. The first 5 cases were reported previously in aggregated form, without molecular characterization of the isolates (*5*). The first 2 patients were young children apparently infected in India in 2003 and 2005. Six additional patients, who were detected in 2006 and 2007, also reported travel to India. Travel to the Indian subcontinent has been associated with nalidixic acid–resistant *S. enterica* serotype Typhi infection; however, ciprofloxacin-resistant infections are rarely reported by using current CLSI criteria (*4**,**5**,**11*). Other resistance patterns were first described in southern Asia, where the incidence of typhoid fever is high and antimicrobial agents are widely available without prescription, providing the opportunity for the development and selection of resistant strains (*8*).

Other than reports by 8 patients of travel to India, we have no information about possible shared exposures, such as specific locations visited, sources of food or water, or contact with carriers of *S. enterica* serotype Typhi. However, the indistinguishable PFGE *Xba*I and *Bln*I patterns and identical *gyrA* and *parC* mutations of isolates from the first 2 patients suggest that, although typhoid fever occurred nearly 2 years apart, the same ciprofloxacin-resistant strain is likely to have been involved. After 2005, different *Xba*I and *Bln*I patterns have been identified in ciprofloxacin-resistant isolates, indicating independent selection of ciprofloxacin resistance in different strains.

The *gyrA* and *parC* mutations of isolates from the first 2 patients were reported in ciprofloxacin-resistant *S. enterica* serotype Typhi in India (*11*). The 2 *gyrA* mutations are well characterized and known to be associated with quinolone resistance; 2 point mutations in *gyrA* and 1 in *parC* confer fluoroquinolone resistance (*8**,**10**–**12*). Further studies, including characterization of other resistance mechanisms, are needed to track the evolution of fluoroquinolone-resistant *S. enterica* serotype Typhi.

Although the ciprofloxacin resistance we detected using current CLSI criteria is rare in *S. enterica* serotype Typhi, nalidixic acid resistance, which correlates with decreased susceptibility to ciprofloxacin, has increased (*7*). Clinicians should be aware that infection with *Salmonella* spp. with decreased susceptibility to ciprofloxacin may not respond satisfactorily to this agent (*6**,**8**,**9**,**13**,**15*). In addition, identification of ciprofloxacin-resistant cases has been increasing. In the presence of quinolone resistance, third-generation cephalosporins, such as ceftriaxone, can be used (*2**,**6**,**8**,**15*). Recent clinical trials suggest that azithromycin might be useful for treating uncomplicated typhoid fever (*2**,**8**,**9**,**15*). Recommendations for empiric treatment of typhoid fever in the United States are best developed by using information about antimicrobial resistance trends in isolates from countries where the infection was acquired.
